# Proteoglycans play a role in the viscoelastic behaviour of the canine cranial cruciate ligament

**DOI:** 10.3389/fbioe.2022.984224

**Published:** 2022-11-15

**Authors:** Rosti Readioff, Brendan Geraghty, Yalda A. Kharaz, Ahmed Elsheikh, Eithne Comerford

**Affiliations:** ^1^ Department of Mechanical, Materials and Aerospace Engineering, School of Engineering, University of Liverpool, Liverpool, United Kingdom; ^2^ Faculty of Engineering, School of Mechanical Engineering, Institute of Medical and Biological Engineering, University of Leeds, Leeds, United Kingdom; ^3^ School of Dentistry, University of Liverpool, Liverpool, United Kingdom; ^4^ Department of Mechanical Engineering, University of Bath, Bath, United Kingdom; ^5^ Institute of Life Course and Medical Sciences, University of Liverpool, Liverpool, United Kingdom; ^6^ Medical Research Council Versus Arthritis Centre for Integrated Research Into Musculoskeletal Ageing (CIMA), University of Liverpool, Liverpool, United Kingdom; ^7^ Beijing Advanced Innovation Center for Biomedical Engineering, Beihang University, Beijing, China; ^8^ NIHR Moorfields BRC, UCL Institute of Ophthalmology, London, United Kingdom; ^9^ School of Veterinary Science, University of Liverpool, Neston, United Kingdom

**Keywords:** knee joint, canine cranial cruciate ligament, proteoglycans (PGs), glycosaminoglycans (GAGs), viscoelasticity, creep, stress relaxation

## Abstract

Proteoglycans (PGs) are minor extracellular matrix proteins, and their contributions to the mechanobiology of complex ligaments such as the cranial cruciate ligament (CCL) have not been determined to date. The CCLs are highly susceptible to injuries, and their extracellular matrix comprises higher PGs content than the other major knee ligaments. Hence these characteristics make CCLs an ideal specimen to use as a model in this study. This study addressed the hypothesis that PGs play a vital role in CCL mechanobiology by determining the biomechanical behaviour at low strain rates before and after altering PGs content. For the first time, this study qualitatively investigated the contribution of PGs to key viscoelastic characteristics, including strain rate dependency, hysteresis, creep and stress relaxation, in canine CCLs. Femur-CCL-tibia specimens (*n* = 6 pairs) were harvested from canine knee joints and categorised into a control group, where PGs were not depleted, and a treated group, where PGs were depleted. Specimens were preconditioned and cyclically loaded to 9.9 N at 0.1, 1 and 10%/min strain rates, followed by creep and stress relaxation tests. Low tensile loads were applied to focus on the toe-region of the stress-strain curves where the non-collagenous extracellular matrix components take significant effect. Biochemical assays were performed on the CCLs to determine PGs and water content. The PG content was ∼19% less in the treated group than in the control group. The qualitative study showed that the stress-strain curves in the treated group were strain rate dependent, similar to the control group. The CCLs in the treated group showed stiffer characteristics than the control group. Hysteresis, creep characteristics (creep strain, creep rate and creep compliance), and stress relaxation values were reduced in the treated group compared to the control group. This study suggests that altering PGs content changes the microstructural organisation of the CCLs, including water molecule contents which can lead to changes in CCL viscoelasticity. The change in mechanical properties of the CCLs may predispose to injury and lead to knee joint osteoarthritis. Future studies should focus on quantitatively identifying the effect of PG on the mechanics of intact knee ligaments across broader demography.

## 1 Introduction

Ligaments are essential to knee joint stability, defined by their material composition, contributing to their complex mechanical characteristics (Girgis et al., 1975; Butler et al., 1978a; Arnoczky, 1983; Frank, 2004). Knee ligaments are strong fibrous tissues consisting of cellular material and extracellular matrix (ECM) proteins such as collagen type I (Frank, 2004). The viscoelastic properties of ligaments are thought to come from the viscous and elastic properties of the collagen fibres (Puxkandl et al., 2002), the interaction of collagen fibres with other non-collagenous components in the ECM such as elastin and proteoglycans (PGs) (Frank, 2004; Henninger et al., 2010, Henninger et al., 2013; Smith et al., 2011, Smith et al., 2014), and water movement (Chimich et al., 1992). In particular, the interactions between sulphated glycosaminoglycans (sGAGs), which are components of proteoglycans (PGs), such as dermatan and chondroitinase sulphate with collagen fibrils in porcine medial collateral ligaments have been reported to increase permeability and decrease peak stress (Henninger et al., 2010).

PGs comprise 0.2%–5% of ligament dry weight and are either non-aggregating [small leucine-rich proteoglycan (SLRPs) such as decorin and biglycan] or large aggregating PGs (versican and aggrecan) (Gillard et al., 1977; Amiel et al., 1984; Hey et al., 1990; Kharaz et al., 2018). They are comprised of a protein core and sulphated GAGs (Vogel, 1994). Approximately 90% of the total PGs in the fresh collateral ligaments are decorin, and the remaining PGs include biglycan, aggrecan, and versican (Ilic et al., 2005). The interactions between PGs with collagen fibrils differ, such that decorin binds to collagen fibrils through its core protein, whereas biglycan and the large PGs, aggrecan and versican, bind to collagen fibrils through their sGAG chains (Pogány et al., 1994; Scott and Thomlinson, 1998; Kharaz et al., 2018). Interactions between the sGAG chains form interfibrillar PG bridges, which are believed to contribute to the mechanical characteristics of collagenous tissues (Scott and Thomlinson, 1998; Scott, 2003). Several studies have shown that decorin contributes to the organisation and mechanical properties of soft tissues such as the skin (Danielson et al., 1997; Eshel and Lanir, 2001; Reed and Iozzo, 2002) and tendons (Robinson et al., 2005, Robinson et al., 2017; Connizzo et al., 2013). The sGAG chains’ role in tissue mechanics has been vital at low stress and strain levels (Eshel and Lanir, 2001; Eckert et al., 2013). Eshel and Lanir (2001) reported that PGs control rat dorsal skin’s response at low strain levels where the collagen fibres were still crimped (toe region of stress-strain behaviour). Similarly, the sGAG content in porcine aortic heart valve leaflets may provide a damping mechanism reducing aortic valve leaflet flutter when the leaflet is not under high tensile stress, reportedly due to strong associations with fibre-fibre and fibre-matrix interaction at low-stress levels (Eckert et al., 2013). In addition to the cross-linking function of sGAG, the chains of sGAG may affect the hydration of soft tissues due to its highly negative charges (Amiel et al., 1995; Thornton et al., 2001; Murienne et al., 2015). The water molecules bond with sGAG can act as a lubricant between collagen fibrils (Scott, 2003) and facilitate the sliding of the fibrils during tensile stretches (Puxkandl et al., 2002), hence affecting the viscoelastic properties of ligaments (Chimich et al., 1992; Thornton et al., 2001; Gautieri et al., 2012).

Most available studies focused on PG’s contribution to articular cartilage material mechanics. Nissinen et al. (2021) and Mäkelä et al. (2015) studied changes in the articular cartilage’s poroelastic material parameters and fluid flow, which are associated with PG content. A computational modelling study representing the heterogeneous internal architecture of cartilaginous tissues such as knee menisci captured the poroelastic behaviour of the tissue (Elmukashfi et al., 2022). However, the previous computational modelling studies on cartilage and other tissues have not explicitly investigated the role of PGs in tissue mechanics nor included ligaments’ microstructure. Hence, investigations of the mechanical role of sGAGs in the knee ligaments are limited. The previous literature focused on the medial collateral ligaments (Lujan et al., 2007, 2009; Henninger et al., 2010). These studies reported that the interactions between sGAGs and collagen fibrils had no impact on the viscoelastic, tensile and resistance properties (Lujan et al., 2007, 2009). However, the permeability of the medial collateral ligament was found to increase with the reduction of sGAG (Henninger et al., 2010), which could be an indication that sGAGs contribute to the mechanical properties of knee ligaments by maintaining tissue hydration. A limitation of these studies was that extracted sections of the ligaments, which might have altered the microstructural organisation of the specimens, were examined instead of testing intact ligaments with their bone attachments *ex vivo* (Henninger et al., 2010).

In the canine knee joint, the cranial cruciate ligament (CCL) is the most susceptible ligament to injury (Moses et al., 2012) and contains a higher PGs content compared to other knee ligaments (Rumian et al., 2007; Kharaz et al., 2018) which likely contribute to the structural integrity of the tissues. Therefore, we hypothesise that a reduction in PGs content would affect the contribution of ECM composition to the structural integrity in the CCL, resulting in altered ligament mechanics, which may ultimately lead to CCL injury and knee joint osteoarthritis (Quasnichka et al., 2005). Thus, this study aimed to qualitatively investigate the role of PGs in the viscoelastic properties (strain rate dependency, hysteresis, creep and stress relaxation) of intact femur-CCL-tibia in an *ex vivo* test environment.

## 2 Materials and methods

### 2.1 Specimen storage, preparation, and purpose

Paired disease-free knee joint cadavers (*n* = 6) from skeletally mature Staffordshire bull terrier canines were obtained with full ethical permission from the Veterinary Research Ethics Committee [(VREC65), University of Liverpool]. Inclusion criteria were knee joints from skeletally mature animals with a bodyweight >20 kg. The entire knee joints were frozen at −20°C until required and defrosted at room temperature for extracting the CCL as a femur-CCL-tibia complex (Readioff, 2017; Readioff et al., 2020a; Readioff et al., 2020b). CCL complexes from the right knee joints were not treated with the PG depletion enzyme (control group), whilst the CCL complexes from the left knee joints were treated to deplete the PGs content (treated group).

### 2.2 Specimen length and cross-sectional area

The CCL lengths were determined between the insertion and origin of the ligaments at the cranial, caudal, lateral and medial planes using Vernier callipers (D00352, Duratool, Taiwan) accurate to ±10 µm (Vasseur et al., 1991; Comerford et al., 2005). The mean values were used in calculating the engineering strains (Readioff, 2017; Readioff et al., 2020b). The method by Goodship and Birch was used to measure the CCLs’ cross-sectional area (CSA) (Goodship and Birch, 2005). In brief, alginate dental impression paste (UnoDent, UnoDent Ltd., United Kingdom) was used to make a mould around the CCL, which was used to create replicas of the ligament. The replicas were cut in half, and the surface of the replicas showing middle CSA was determined using ImageJ (a public domain Java image processing program) (Readioff, 2017; Readioff et al., 2020b). The CSA values were then used in the calculations of engineering stress.

### 2.3 Chondroitinase treatment protocol

The CCLs from both groups were immersed for 1 h at room temperature (20°C) in 20 ml buffer solution (15 ml of 20 mM Tris pH 7.5, 150 mM NaCl, 5 mM CaCl2) with protease inhibitors (1 tablet of mini-cOmplete per 10 ml of buffer, SIGMA-ALDRICH/Roche, United States) (Lujan et al., 2009). Chondroitinase ABC (ChABC) 0.25 IU/ml (SIGMA-ALDRICH, United States) enzyme was dissolved in 0.01% bovine serum albumin (BSA), and samples were incubated in this solution for 3 h prior to the mechanical tests as previously described (Lujan et al., 2009). This process was performed to reduce PG contents, and it was based on our preliminary work. Our preliminary work showed that approximately 80% of PGs in sectioned CCLs were digested within 3 h of incubation [Supplementary Materials (Supplementary Table S1 and Supplementary Figure S1)]. Control and treated groups were preserved during the mechanical tests in a custom-built tank filled with 600 ml of the buffer solution and protease inhibitors at room temperature (20°C) (1 tablet of cOmplete Protease Inhibitor Cocktail per 50 ml of buffer, SIGMA-ALDRICH/Roche, United States).

### 2.4 Mechanical testing protocol

Each femur-CCL-tibia complex examined was attached to an Instron 3,366 (Instron, Norwood, MA) material testing machine fitted with a 10 N load cell (Instron 2,530–428 with ±0.025 N accuracy) using a custom-built stainless steel ducktail clamp and rig (Readioff et al., 2020a; Readioff et al., 2020b). A pre-load of 0.1 N was applied, followed by five load-unload preconditioning cycles to a maximum load of 9.9 N at a 10%/min strain rate (Butler et al., 1978b; Fung, 1993; Savelberg et al., 1993; Provenzano et al., 2002). Subsequently, mechanical tests were performed examining strain rate, creep and stress relaxation behaviours. The strain rate tests consisted of 1) two loading cycles at 0.1%/min strain rate; 2) three loading cycles at 1%/min strain rate; and 3) two loading cycles at 10%/min strain rate. These loading cycles were successively applied, followed by two cycles for creep testing and two for stress relaxation testing.

The creep behaviour of the CCLs was determined by subjecting the ligament to tensile loads of 4.9 N and 9.9 N that remained constant for 15 min each. For the stress relaxation tests, the CCLs were extended by applying a 9.9 N tensile load and monitoring the gradual decrease in tissue stress over 15 min whilst the ligament extension was held constant. Loading and unloading during creep and stress relaxation tests were performed at a 1%/min strain rate. A recovery period of 6 min was applied between each loading-unloading cycle to minimise the effect of the strain history of previous cycles on subsequent behaviour (Readioff et al., 2020a; Readioff et al., 2020b).

Following completion of the mechanical tests, the middle section of each CCL was extracted in preparation for the biochemical assays to determine water and sGAG contents in both control and treated groups (Farndale et al., 1986).

### 2.5 Biochemical assays

#### 2.5.1 sGAG content quantification

CCLs in control and treated groups were digested for 48 h with 10 unit/ml papain in 100 mM sodium acetate, 2.4 mM ethylenediaminetetraacetic acid (EDTA), and 5 mM cysteine hydrochloric acid (HCL) at 60°C (Farndale et al., 1986). Dimethylmethylene blue (DMMB) dye-binding assay (1, 9-dimethylmethylene blue) was used to determine the sGAG content of the CCLs (Farndale et al., 1986; Kharaz et al., 2018). Subsequently, 250 µl of DMMB dye was added to 40 µl duplicates of papain-digested CCLs, and this was immediately analysed at 570 nm wavelength. Shark chondroitin sulphate over a concentration range of 0–75 μg/ml was used as a standard, and sGAG content was calculated by comparison with the standard line (Lujan et al., 2009; Kharaz et al., 2018).

#### 2.5.2 Water content quantification

The water content of the CCL in both groups was expressed in terms of the mass of water per unit mass of the wet ligament (Eq. 1) as described previously (Lujan et al., 2009; Kharaz, 2015). Initially, the CCLs were left to thaw at room temperature (20°C), and wet mass was measured. Subsequently, these samples were freeze-dried overnight, and then the dry masses of the CCLs were measured.

$$Water\;Content\;\left(\%\right)=\frac{Wet\;mass-Dry\;mass}{Wet\;mass}\times 100$$ (1)

### 2.6 Viscoelastic data analysis

Analyses of the load-deformation data were performed using MATLAB (MATLAB R2020b), and the mean and standard deviation of the analysed data were reported. Eqs 2, 3 were used to calculate engineering stress and strain values (Haut and Little, 1969; Woo et al., 1981). Subsequently, the secant modulus for the maximum applied stress describing the stiffness of the CCLs was determined (Eq. 4). Numerical integration (using the trapezoidal rule) of the load-unload stress-strain curves was used to estimate the stored energy in the ligaments (Eq. 5). The hysteresis (dissipated energy) was then calculated from the difference between the stored energy during loading and unloading cycles (Elsheikh et al., 2008) (Eq. 6). Creep behaviour was determined from the strain-time curves and creep compliance function (Eq. 7). The stress relaxation behaviour was determined from the stress-time curves and stress relaxation modulus (Eq. 8). Stress values were normalised by the peak stress at the test start time (*t* = 0), allowing the comparison of relaxation behaviour across ligaments (Fung, 1993). The stress relaxation rate was calculated at 15 min.

$$\sigma =\frac{F}{CSA}$$ (2)

where σ is stress in MPa, F is applied load in N, and CSA is the cross-sectional area at the middle of the CCL in mm^2^.

$$\varepsilon =\frac{∆L}{L_{0}}$$ (3)

where ε is strain, ∆L is the change in length in mm ( $∆L=L_{1}-L_{0}$ ), and $L_{0}$ is initial length and $L_{1}$ is the deformed length of the CCL in mm.

$$E_{secant}=\frac{\sigma_{\max}}{\varepsilon_{\max}}$$ (4)

where $E_{secant}$ is secant modulus in MPa, and $\sigma_{\max}$ and $\varepsilon_{\max}$ are the maximum stress and strain values of the load cycle.

$$U=\overset{N}{\underset{k=1}{\sum }}\frac{1}{2}\times \left(\sigma_{k-1}+\sigma_{k}\right)\times ∆\varepsilon_{k}$$ (5)

where U is the stored energy in MPa, N is the resolution of the trapezoidal partition, and $∆\varepsilon_{k}$ is the length of the $k^{th}$ interval ( $∆\varepsilon_{k}=\varepsilon_{k}-\varepsilon_{k-1}$ ).

$$Hysteresis=U_{Loading}-U_{Unloading}$$ (6)

where $U_{Loading}$ and $U_{Unloading}$ represent the stored energy during the loading and unloading of the ligaments, respectively, in MPa.

$$J\left(t\right)=\frac{\varepsilon \left(t\right)}{\sigma_{0}}$$ (7)

where J(t) is the creep compliance function in MPa^−1^, and $\sigma_{0}$ is the initial applied stress.

$$G\left(t\right)=\frac{\sigma \left(t\right)}{\varepsilon_{0}}$$ (8)

where G(t) is the relaxation modulus function in MPa, and $\varepsilon_{0}$ is the initial applied strain.

## 3 Results

### 3.1 Specimen characteristics

The CCL specimens (*n* = 6 paired knee joints) were of mixed gender (female = 1 and male = 5), and the bodyweight of the cadavers was in the range of 21.5–29.4 kg (mean ± standard deviation: 25.76 ± 3.12 kg).

### 3.2 Specimen length and cross-sectional area

The CCLs’ mean lengths and CSA ranged from 14.58 to 19.25 mm (mean ± standard deviation: 16.42 ± 1.33 mm) and from 16.07 to 31.57 mm^2^ (mean ± standard deviation: 23.79 ± 5.08 mm^2^), respectively. The length and CSA of each CCL can be found in the Supplementary Tables S2, S3).

### 3.3 Biochemical assays

#### 3.3.1 sGAG content

The depletion process reduced sGAGs by approximately 19% in the treated group (Table 1). The sGAG content of the CCLs in the treated group ranged from 1.7 to 4.7% (mean ± standard deviation: 3.1 ± 1.1%) as a percentage of dry weight, whereas the range was 2.2–6.6% (mean ± standard deviation: 3.8 ± 1.6%) in the control group.

**TABLE 1:** The sulphated glycosaminoglycan (sGAG) contents (micrograms of sGAG per milligram ligament dry weight) of the cranial cruciate ligaments (CCLs) in control (proteoglycans were not depleted) and treated (proteoglycans were depleted) groups.

CCL specimens	Micrograms of sGAG per milligram dry weight of the CCL (%)	
	Control group	Treated group
Specimen 1	3.8	2.5
Specimen 2	6.6	4.7
Specimen 3	2.2	1.7
Specimen 4	2.2	2.2
Specimen 5	3.9	3.5
Specimen 6	4.0	3.8
Mean	3.8	3.1
Standard Deviation	1.6	1.1

#### 3.3.2 Water content

The depletion process reduced water content by approximately 4% in the treated group (Table 2). The water content of the CCLs in the treated group ranged from 65.7 to 73.3% (mean ± standard deviation: 69.4 ± 3.0%), whereas the range was 64.7–77.4% (mean ± standard deviation: 72.3 ± 4.2%) in the control group.

**TABLE 2:** The water content of the cranial cruciate ligaments (CCLs) in control (proteoglycans were not depleted) and treated (proteoglycans were depleted) groups.

CCL specimens	Water content (%)	
	Control group	Treated group
Specimen 1	77.4	71.3
Specimen 2	74.2	71.3
Specimen 3	73.2	68.6
Specimen 4	71.9	73.3
Specimen 5	72.5	65.7
Specimen 6	64.7	66.5
Mean	72.3	69.4
Standard Deviation	4.2	3.0

### 3.4 Mechanical properties

#### 3.4.1 Stress-strain

**FIGURE 1:**
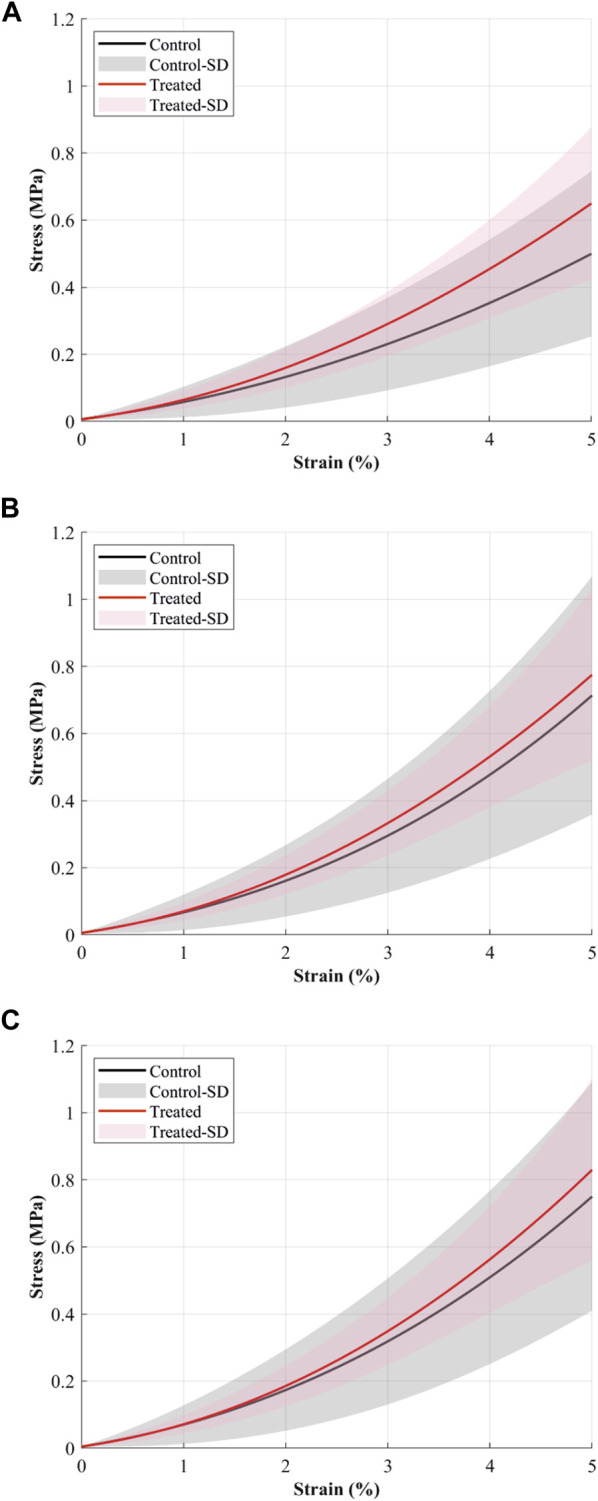
The mean stress-strain curves (solid continuous lines) of cranial cruciate ligaments (CCL) at **(A)** 0.1%/min, **(B)** 1%/min, and **(C)** 10%/min strain rates during loading tests. The black line and grey shaded standard deviation show results from the control (proteoglycans were not depleted) group, and the red line and light red shaded standard deviation show results from the contralateral CCLs in the treated (proteoglycans were depleted) group.

The experimental setup was designed to focus on the toe-region of the stress-strain curves where the extracellular matrix, including the PGs, is expected to affect the CCL mechanics (Eshel and Lanir, 2001; Lujan et al., 2009; Henninger et al., 2010; Eckert et al., 2013). The stress-strain behaviour of the CCLs in control and treated groups followed a similar pattern, showing strain rate dependencies (Figures 1A–C). For example, the stress-strain behaviour of specimens in both groups showed an increase in stiffness with increasing strain rates from 0.1 to 1 and then to 10%/min.

The CCLs in the treated group illustrate higher stress values than those in the control group. During loading at 0.1%/min strain rate, the mean stress at 5% strain was 0.5 MPa in the control and 0.65 MPa in the treated groups (Figure 1A). Similar patterns were found during loading at 1 and 10%/min strain rates. During 1%/min strain rate, the mean stress at 5% strain was 0.71 MPa in the control and 0.77 MPa in the treated groups (Figure 1B); and during 10%/min strain rate, the mean stress at 5% strain was 0.75 MPa in the control and 0.83 MPa in the treated groups (Figure 1C).

#### 3.4.2 Secant modulus

**FIGURE 2:**
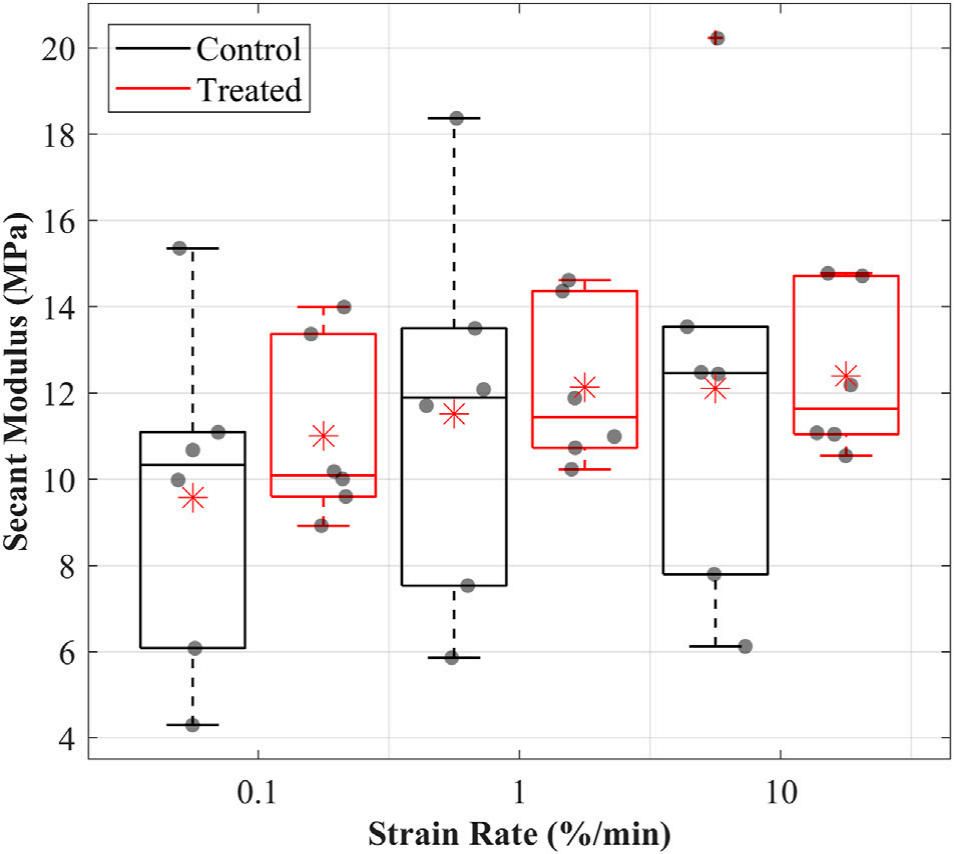
Secant modulus values were determined for the cranial cruciate ligaments (CCLs) in the control (black line) group, where proteoglycans were not depleted, and the treated (red line) group, where proteoglycans were depleted, at varying strain rates. The box plot shows individual specimen values (grey dots) and means (red asterisk) during loading at 0.1%/min, 1%/min, and 10%/min strain rates. The outliers are indicated with a red plus sign.

The secant moduli for the maximum applied stress of the CCLs increased with increasing strain rate in both groups (Figure 2). Similar to the patterns observed in the stress-strain behaviour, the mean secant moduli were higher in the treated than in the control groups. At 0.1%/min strain rate, the mean secant moduli were 9.6 MPa in the control and 11 MPa in the treated groups. Similar patterns were found during loading at 1 and 10%/min strain rates. At 1%/min strain rate, the mean secant moduli were 11.5 MPa in the control and 12.2 MPa in the treated groups, while at 10%/min strain rate, the mean secant moduli were 12.1 MPa in the control and 12.4 MPa in the treated groups.

#### 3.4.3 Hysteresis

**FIGURE 3:**
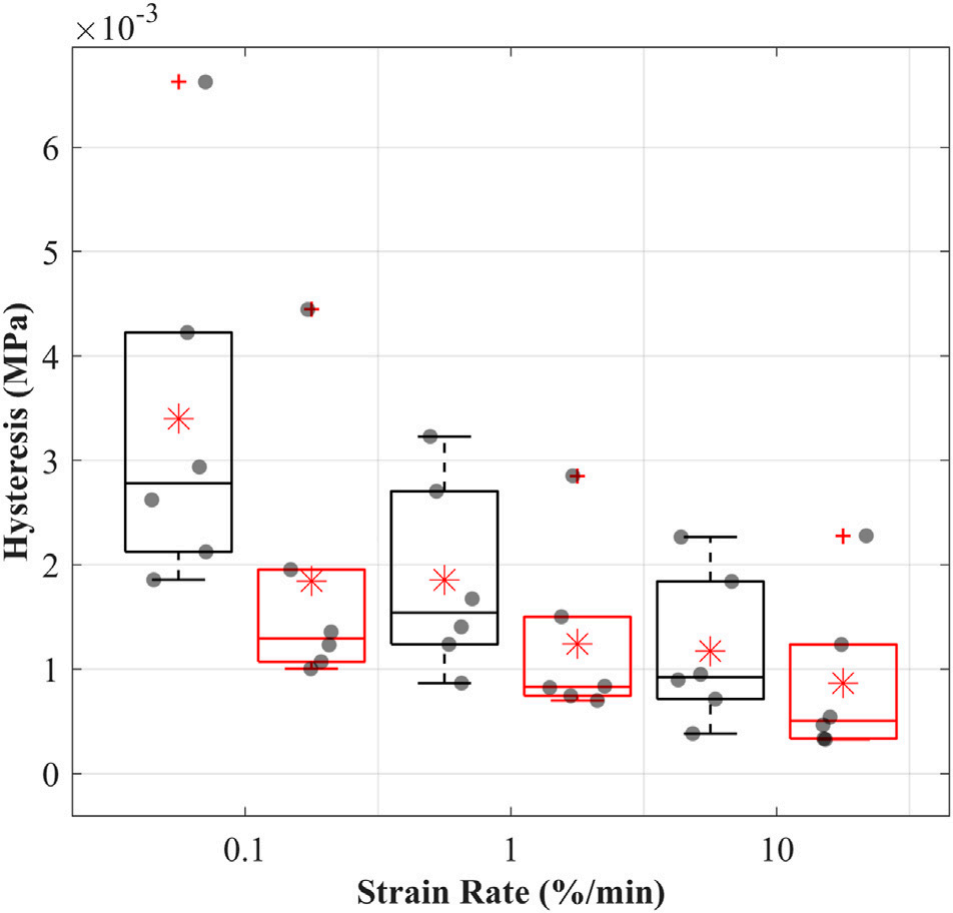
Hysteresis (dissipated energy) of the cranial cruciate ligaments (CCLs) during cyclic loading at varying strain rates for the control (black lines) group, where proteoglycans were not depleted, and the treated (red lines) group, where proteoglycans were depleted. The box plot shows individual specimen values (grey dots) and means (red asterisk) during loading at 0.1%/min, 1%/min, and 10%/min strain rates. The outliers are indicated with a red plus sign.

The hysteresis decreased with increasing strain rates, suggesting strain rate dependencies of the CCLs in both groups (Figure 3). Hysteresis was consistently higher in the control than in the treated group across all three strain rates. The mean hysteresis at 0.1, 1, and 10%/min were 3.4, 1.8, and 1.2 MPa in the control and 1.8, 1.2, and 0.8 MPa in the treated groups.

#### 3.4.4 Creep

**FIGURE 4:**
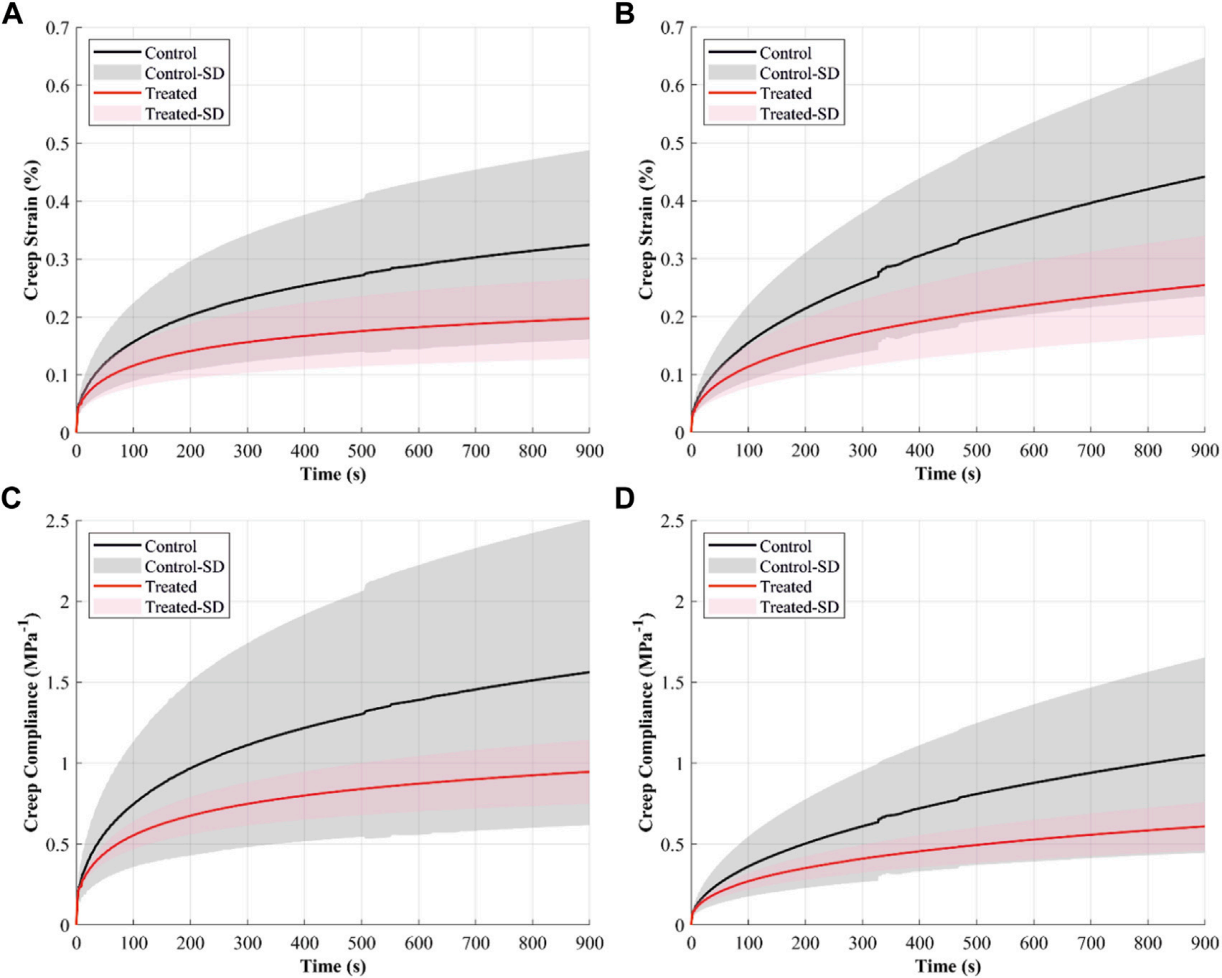
The creep behaviour of the cranial cruciate ligaments (CCLs) shows creep strain-time curves at **(A)** 4.9 N and **(B)** 9.9 N applied loads and creep compliance curves at **(C)** 4.9 N and **(D)** 9.9 N applied loads. The black line (mean) and the grey shade (standard deviation) show results from the control group (proteoglycans were not depleted), and the red line (mean) and light red shade (standard deviation) show results from the contralateral CCLs in the treated group (proteoglycans were depleted).

The creep strain-time curves showed increased strain with time in both control and treated groups (Figures 4A,B). The creep strains were higher in the control than in the treated groups during creep loads of 4.9 and 9.9 N. For example, after 15 min of 4.9 N creep load, the mean creep strain was recorded at 0.32% in the control and 0.2% in the treated groups.

Creep compliance, indicating strain per unit stress, was higher in both groups when the CCLs were subjected to a constant creep load of 4.9 N than 9.9 N (Figures 4C,D). The CCLs in the control showed higher creep compliance than the treated group during loading at 4.9 and 9.9 N.

**FIGURE 5:**
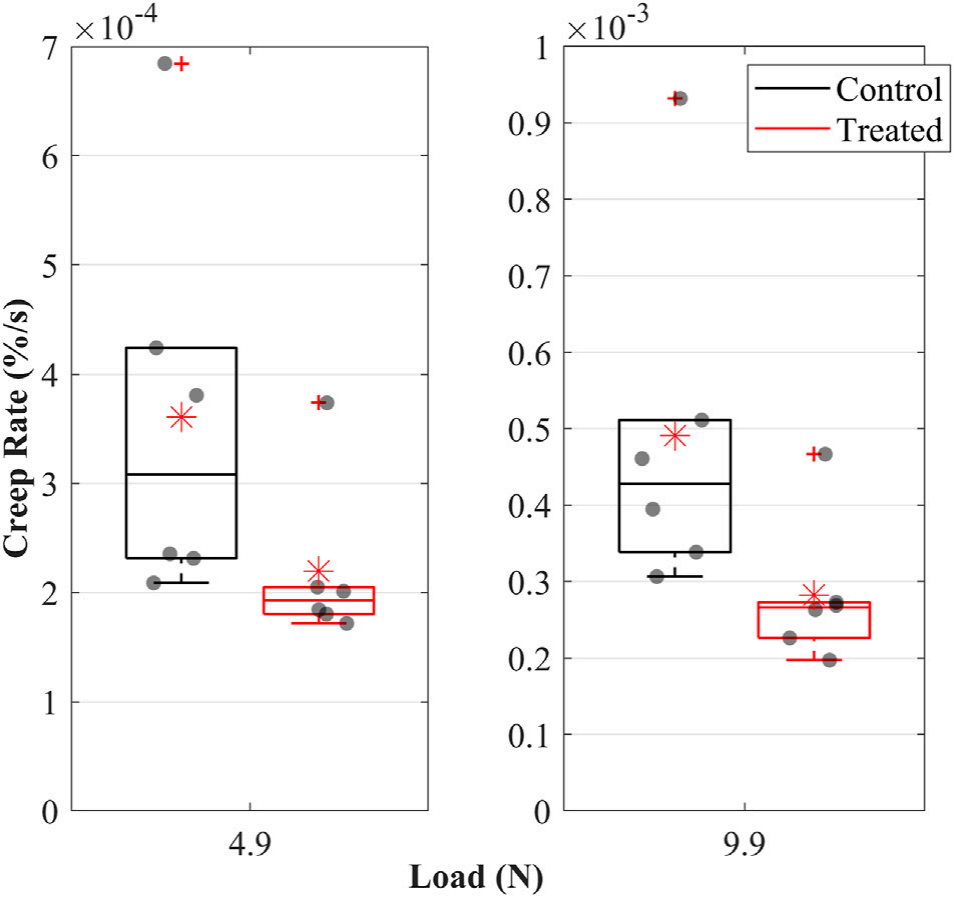
Creep rate of the cranial cruciate ligaments (CCLs) during loading at 4.9 N and 9.9 N in the control (black lines) group, where proteoglycans were not depleted, and in the treated (red lines) group, where proteoglycans were depleted. The box plot shows individual specimen values (grey dots) and means (red asterisk). The outliers are indicated with a red plus sign.

The creep strain rate increased when the load was increased from 4.9 to 9.9 N; this was evident in both groups (Figure 5). Creep strain rates were higher in the control than in the treated groups. During constant loads of 4.9 and 9.9 N, mean creep rates were 0.36 × 10^−3^ and 0.5 × 10^−3^% in the control and 0.22 × 10^−3^ and 0.3 × 10^−3^% in the treated groups.

#### 3.4.5 Stress relaxation

**FIGURE 6:**
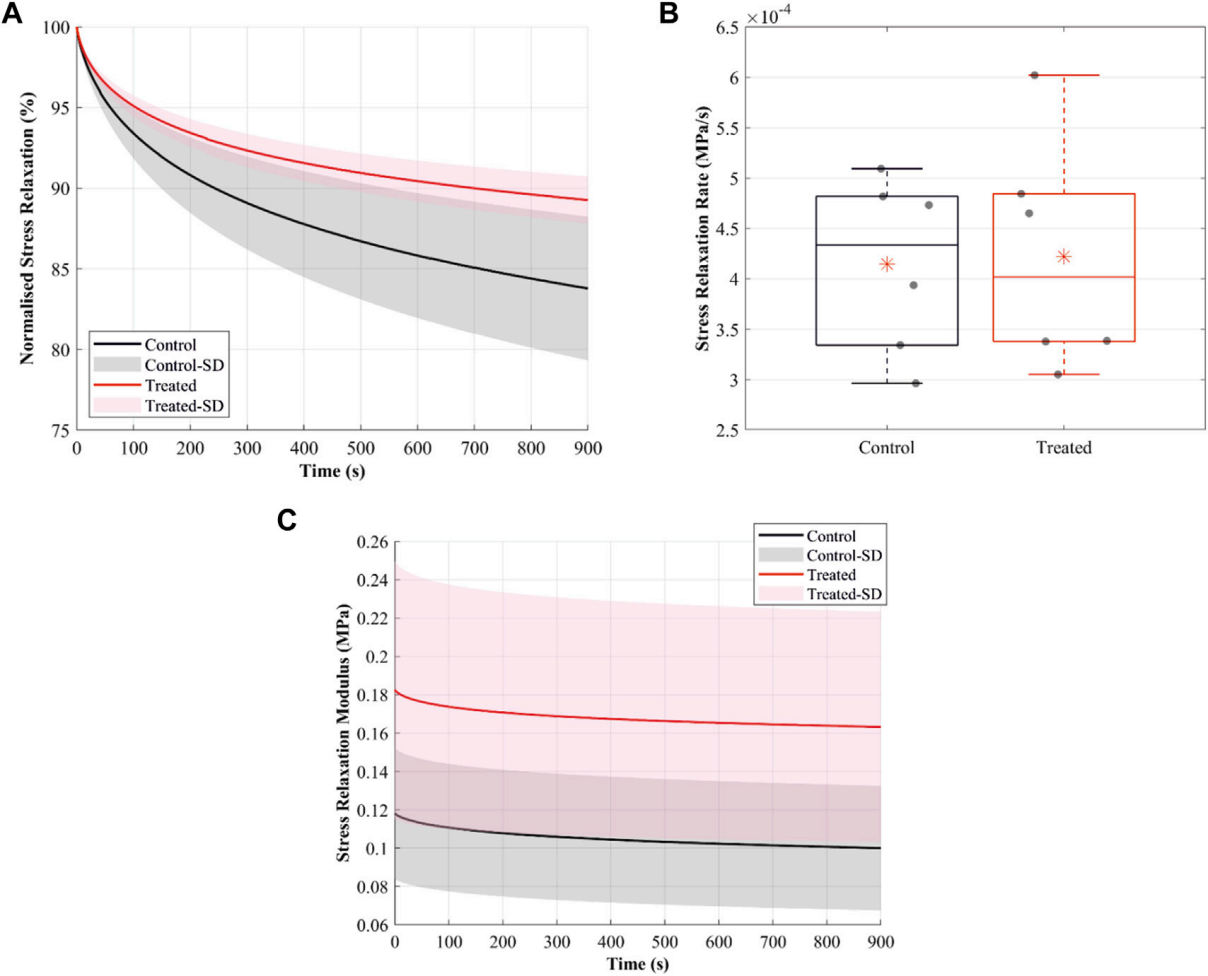
Stress-relaxation behaviour of the cranial cruciate ligaments (CCLs) showing **(A)** normalised stress relaxation-time curves in the control (black lines: mean, grey shade: standard deviation) group, where proteoglycans were not depleted, and in the treated (red lines: mean, light red shade: standard deviation) group, where proteoglycans were depleted. **(B)** Stress relaxation rate in the control (black lines) and the treated (red lines) groups. The box plot shows individual specimen values (grey dots) and means (red asterisk). The outliers are indicated with a red plus sign. **(C)** Stress relaxation modulus in the control (black lines: mean, grey shade: standard deviation) and the treated (red lines: mean, light red shade: standard deviation) groups.

The normalised stress relaxation-time curves illustrate greater stress relaxation in the control group than in the treated groups (Figure 6A). For example, at 15 min of relaxation, the mean values for normalised stress relaxation were 84% in the control and 89% in the treated groups. However, the stress relaxation rate was slightly lower in the control than in the treated groups, with the mean values being 0.41 × 10^−3^ MPa/s in the control and 0.42 × 10^−3^ MPa/s in the treated groups (Figure 6B).

The stress relaxation modulus, indicating stress variations under the imposed constant unit strain, was higher in the treated than control groups (Figure 6C). The mean values for the relaxation modulus at *t* = 0 were 0.12 MPa in the control and 0.18 MPa in the treated groups.

## 4 Discussion

This study focused on qualitatively observing and describing changes in the viscoelastic characteristics of the CCLs due to changes in the PG content. We hypothesised that altering PGs content in the CCLs, which changes the composition of the ligaments, might affect the viscoelastic characteristics of the CCL. This compositional change is clinically significant because it can affect ligament mechanics, possibly predisposing to a CCL injury and knee joint osteoarthritis (Quasnichka et al., 2005). Therefore, this study qualitatively analysed the contribution of PGs to the viscoelastic behaviour of intact femur-CCL-tibia, particularly the role of PGs in the strain rate dependency, hysteresis, creep, and stress relaxation of the CCLs. Here, we found alterations in the viscoelastic characteristics of the CCLs due to PGs reduction. Changes were observed in tissue stiffness, hysteresis, stress relaxation and creep. However, strain rates and the increase in creep load were unaffected by the PG content reduction.

The design of experiments and mechanical tests were performed based on our previous work (Readioff et al., 2020a; Readioff et al., 2020b) and preliminary investigations (Comerford et al., 2014). The mechanical tests focused on investigating the toe-region of the stress-strain curves, where the collagen fibres are crimped, and sGAG chains are believed to have a significant mechanical contribution to tissues (Eshel and Lanir, 2001; Lujan et al., 2009; Eckert et al., 2013), hence in this study, we examined loads up to 10 N at three slow strain rates (Haut and Little, 1969; Readioff et al., 2020b). The ligaments were not loaded to failure as this study focused on the toe-region of the stress-strain curves. Additionally, the loading protocol optimised the use of specimens for multiple studies, including the regional distribution of PGs across the ligament (Kharaz et al., 2018). Future studies could explicitly investigate the sensitivity of PGs content to the mechanics of the ligaments during higher loading conditions, including failure loads.

The CCL properties such as length and the cross-sectional area in the mid-ligament regions were determined and used in the calculations of engineering stress and strain values, and these properties were in a range similar to those previously reported for canine CCLs (Butler et al., 1983; Comerford et al., 2005).

When determining the incubation process of the CCLs in chondroitinase ABC (ChABC), our preliminary time-course study showed that after 3 h of incubation in 0.25 IU/ml ChABC, sGAG content was significantly reduced by approximately 82.3% [Supplementary Materials (Supplementary Figure S1)] similar to previous studies (Lujan et al., 2007, 2009; Henninger et al., 2010). However, unlike the current study, where intact femur-CCL-tibia complexes were used, the preliminary investigation was carried out on CCLs that were transversely cut to extract their middle sections. The transverse cut of the CCLs in the preliminary investigation might have disrupted the synovial sheath allowing better infiltration of the enzyme and reducing the sGAG content. The CCL is surrounded by vascularised synovial tissue (synovial sheath), which protects the ligament’s core tissue from exposure to synovial fluid and hence degradation (Amiel, 1990; Petersen and Tillmann, 1999; Chen et al., 2019). The CCLs can be described as fibre-reinforced matrices, and approximately 70% of the CCLs are water. The water content in the CCLs is associated with sGAG chains, and these chains bind with water molecules because of their highly negative charges (Amiel et al., 1995; Thornton et al., 2001; Murienne et al., 2015). In this study, reducing PG content reduced water content by approximately 4%. The change in water content may indicate that when the sGAG chains were reduced, the matrix lost water-binding sites, reducing CCL’s water-retaining capacity.

The stress-strain behaviour of the CCLs was unaffected by the reduction of the PGs content (Figure 1), including strain rate dependency. This outcome agrees with the human medial collateral ligament study, where the removal of dermatan sulphate showed no effect on the quasi-static tensile property (Lujan et al., 2007). Previous studies reported stress-strain behaviour of untreated CCLs under strain rates ranging from 0.1 to 36.8%/min (Haut and Little, 1969; Readioff et al., 2020b). Stresses at 5% strain were 2.1 MPa at 1.7%/min, 2.5 MPa at 2.6%/min, and 3.5 MPa at 10.8%/min (Haut and Little, 1969), and they were higher than the results reported in this study. For example, stresses of the CCLs in the control group at 5% strain ranged between 0.5 and 0.75 MPa at strain rates up to 10%/min. However, stresses in human ACLs at 5% strain was approximately 0.05 MPa in an old osteoarthritic knee when mechanically tested at 1,400%/min (Peters et al., 2022), and it is lower than reported values in this study, possibly due to the joint degeneration effect on the ligaments.

The secant modulus, showing CCL stiffness, was higher in the treated than control groups (Figure 2). The increase in secant modulus may be linked with the decrease in water content due to changes in the sGAG chains. The decrease in sGAG chains reduces the water-binding sites, reducing water molecules and means for tissue lubrication, increasing internal friction and tissue stiffness (Puxkandl et al., 2002; Scott, 2003; Legerlotz et al., 2013). The modulus of untreated CCLs has been reported as approximately 260 MPa under failure load at a 6,000%/min strain rate equivalent to 2.3 MPa at 0.5% of the applied stress (Butler et al., 1983). The equivalent modulus is lower than the secant moduli reported in this paper for the CCLs in the control group, and they ranged between 10 and 12 MPa at strain rates up to 10%/min. The marked variability in the reported values is likely due to variations in testing techniques and specimen demographics, making it challenging to perform comprehensive comparisons.

Hysteresis of the CCLs in both groups was strain rate dependent, and this characteristic did not change after PGs reduction (Lujan et al., 2007; Readioff et al., 2020b). The treated CCLs had lower hysteresis than those in the control group, suggesting that PG depletion has altered the microstructural organisation of the tissue affecting energy storage (Figure 3). The mean hystereses in the control group were approximately 3.5, 1.8, and 1.2 MPa at 0.1, 1 and 10%/min, and these values are comparable to the previously reported hysteresis ranging from three to 1.5 MPa at strain rates up to 10%/min where similar test methodologies were adopted (Readioff et al., 2020b).

The decrease in creep after PG reduction (Figures 4, 5) can result from decreased hydration (Thornton et al., 2001). With higher water molecules inside the tissue, the creep compliance is increased because water provides greater freedom for fibrillar movement (Thornton et al., 2001). Similarly, Murienne et al. (2015) associated the slower creep rate with increased interfibrillar friction with sGAG removal.

Reducing sGAG chains reduced normalised stress relaxation (Figure 6A), and at the low strain level, stress relaxation likely occurred through sliding between collagen fibres (Screen et al., 2013). The results in this study support the notion that the higher water content in the control group permits greater relative movement and, hence, greater relaxation of the microstructural component in the ligaments (Chimich et al., 1992). Larger and faster stress relaxation was observed in mouse tail tendon decorin knockout (Elliott et al., 2003) and mice tendon fascicles (Legerlotz et al., 2013). However, Lujan et al. (2009) showed a small and negligible increase in stress relaxation after reducing sGAG in the human medial collateral ligament. In this study, the change in the relaxation behaviour after treatment can cause fatigue damage in the CCLs, highlighting the critical role of sGAG in tissue mechanics, possibly by maintaining ligament hydration.

Our study had several limitations, one of which was the small reduction in PGs in the intact femur-CCL-tibia complexes. Future studies could overcome this limitation by injecting the enzyme into the intact femur-CCL-tibia complexes to reduce PGs content further. This process could be adapted from the current practice for reducing PGs in cadaveric articular cartilage (Dixon et al., 2021; Culbert et al., 2022). Future work could also image the pattern of collage-proteoglycan interaction in the ligaments using previously established methods (Scott, 1985; Raspanti et al., 2000) and focus on such interactions under dynamic loading conditions.

The approximation methods adopted to measure the cross-sectional area and length of the CCLs might be another limitation. However, these methods were selected because of their non-destructive approach. Further investigation with a larger number of specimens will allow for quantitative (statistical) measures and an improved understanding of the effect of cadaveric demography (i.e., age, gender and body weight) on the mechanical properties of the CCLs (Woo et al., 1990a; Woo et al., 1990b; Duval et al., 1999).

In conclusion, to the authors’ knowledge, this study is the first to qualitatively describe the contribution of PGs to key viscoelastic characteristics of the intact femur-CCL-tibia complex in canine knee joints. We have shown that reducing sGAG chains in the CCLs increases stiffness and stress relaxation modulus and decreases creep strain, creep compliance and creep rate. However, strain rate sensitivity and the sensitivity to the increase in creep load were unaffected by the reduction of the sGAG chains. This study lacks a sufficient sample size and, consequently, lacks a statistical analysis to conclude the significance of the results. Our results suggest a tendency and not a significant behaviour that the role of sGAG is essential in maintaining microstructural organisation, including water molecules in the tissue, which in effect contributes to the viscoelasticity of the CCLs and, ultimately, knee joint stability.

## Data Availability

The original contributions presented in the study are included in the article’s Supplementary Materials, further inquiries can be directed to the corresponding authors.
