# Supplementary material for: Proteoglycans play a role in the viscoelastic behaviour of the canine cranial cruciate ligament
Source: Front Bioeng Biotechnol. 2022 Nov 15;10:984224. doi: 10.3389/fbioe.2022.984224 (PMC9705345; doi:10.3389/fbioe.2022.984224)
Supplement: Supplementary file 3 [file DataSheet1.docx]

Supplementary Material

# Supplementary Data

Supplementary data are uploaded separately.

# Supplementary Figures and Tables

## Supplementary Figures

|  |
| --- |

Figure S1: The figure shows a decrease in sulphated glycosaminoglycan (sGAG) content (%) in the cranial cruciate ligaments (CCLs) with increasing incubation time for four chondroitinase ABC (ChABC) concentration levels (Supplementary Materials (Table S1)). Results indicate that after three hours incubation in 0.25IU/ml ChABC, approximately 82.3% of sGAG is depleted.

| **0.1%/min – control** | 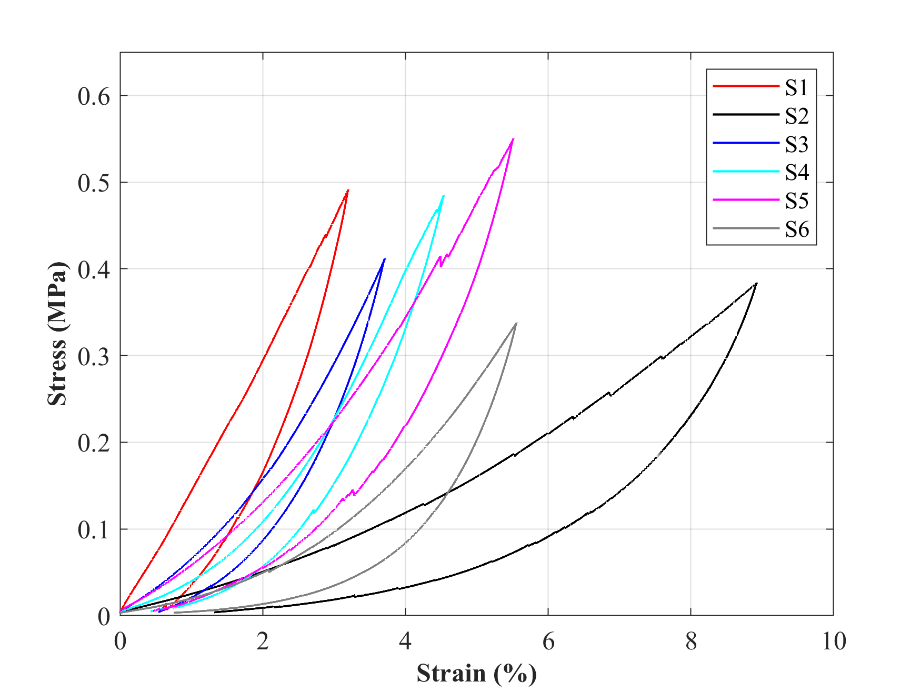 |
| --- | --- |
| **0.1%/min – treated** | 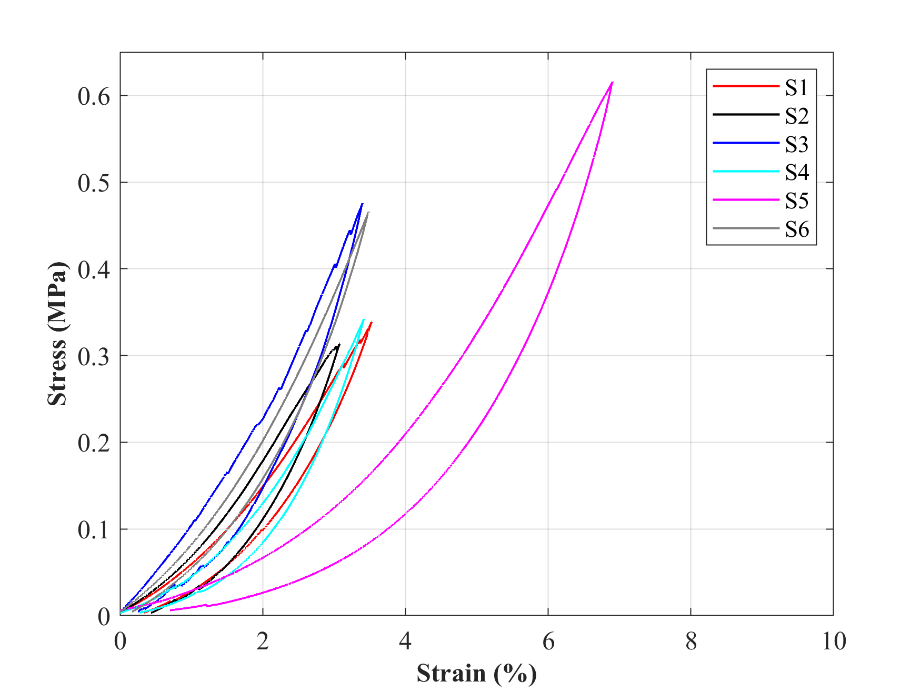 |
| **1%/min – control** | 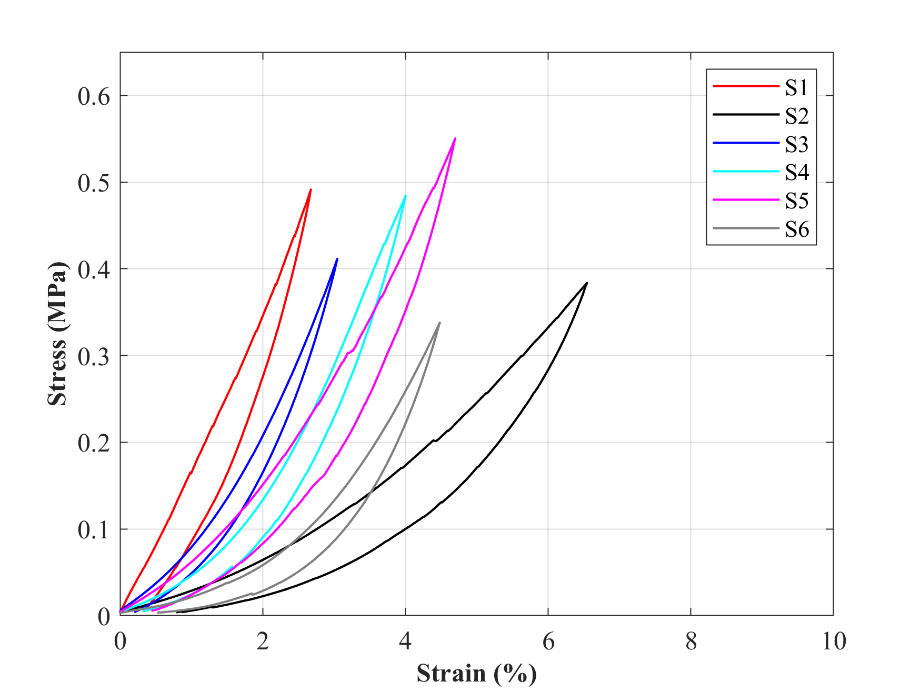 |
| **1%/min – treated** | 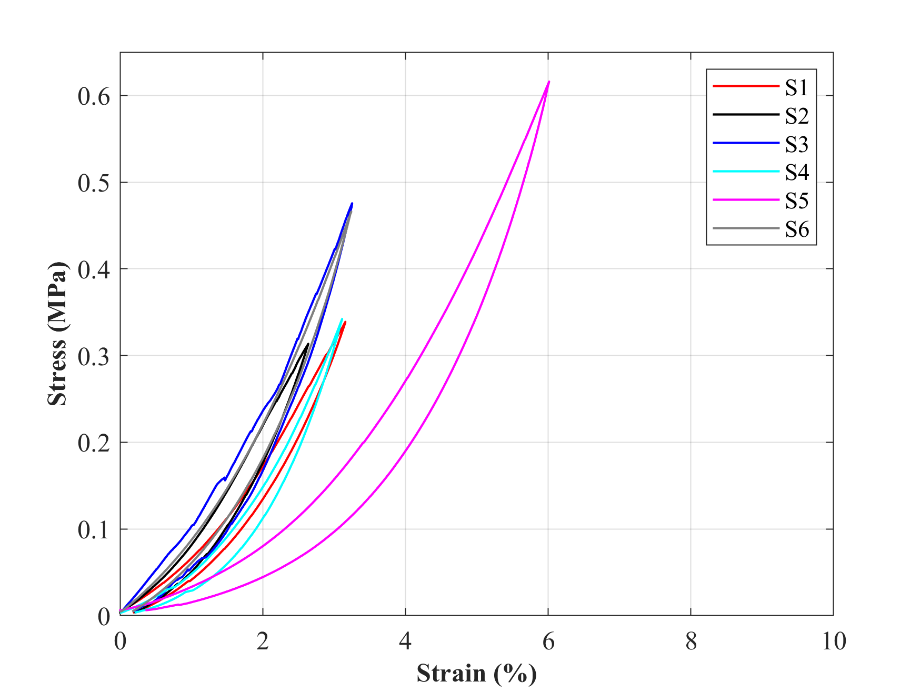 |
| **10%/min – control** | 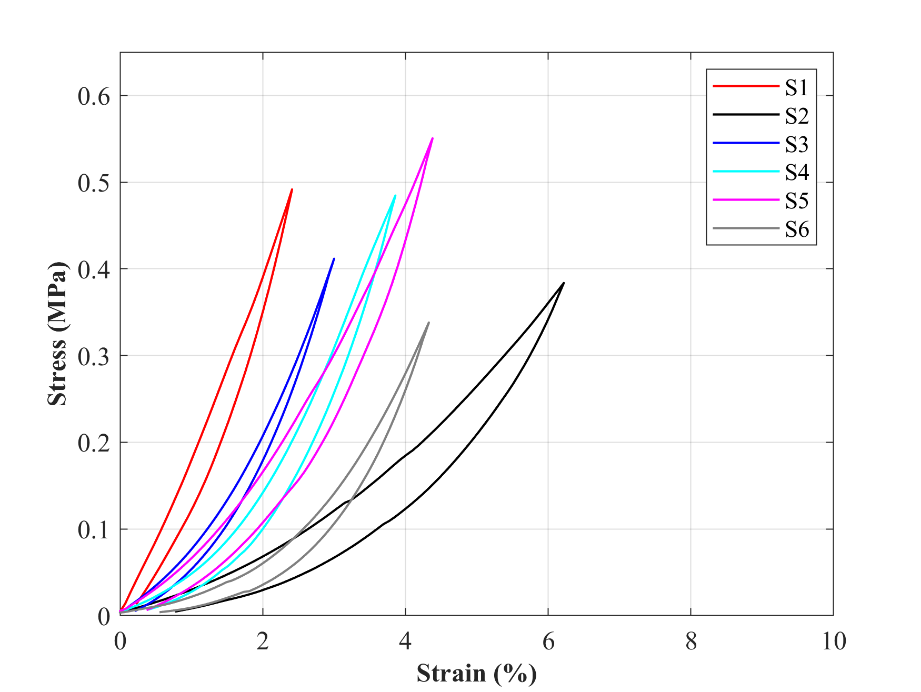 |
| **10%/min – treated** | 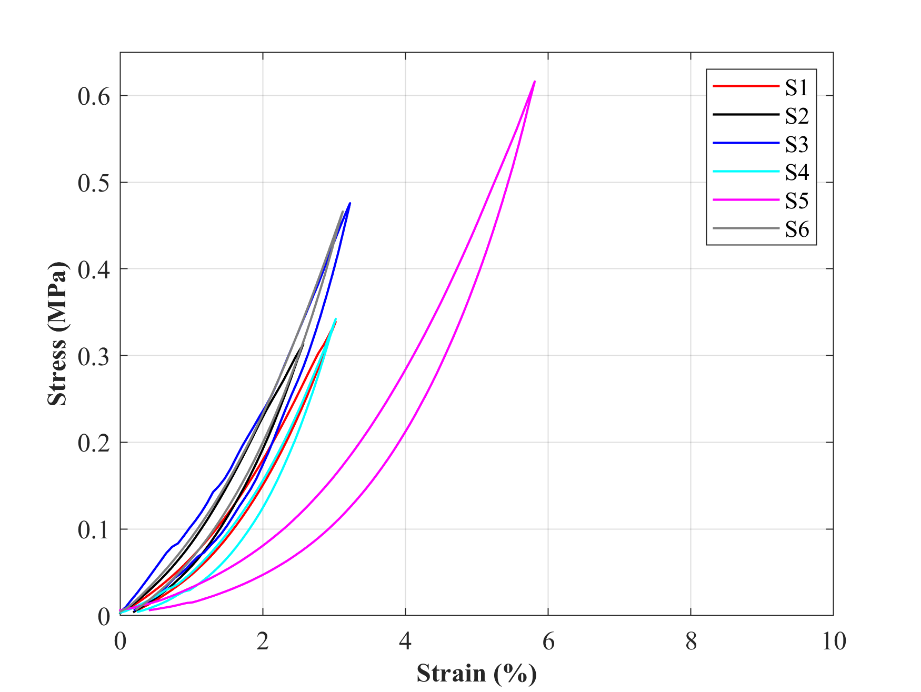 |

Figure S2: Stress-strain behaviour of canine cranial cruciate ligaments (CCLs) during load and unload tensile tests in the control and treated (with PG - depletion) groups at 0.1, 1 and 10 %/min strain-rates.

## Supplementary Tables

Table S1: This table shows results of a preliminary time-course study on reduction of sulphated glycosaminoglycans (sGAG) in canine cranial cruciate ligaments (CCLs). The measurements are for wet and dry weight (mg), water content (%), and sGAG content relative to dry weight of the CCLs. Abbreviations: IU, International Unit.

| **CCL groups** | **Wet weight (mg)** | **Dry weight (mg)** | **Water content (%)** | **Microgram of sGAG/mg dry weight** | **Microgram sGAG/mg dry weight (%)** |
| --- | --- | --- | --- | --- | --- |
| **Control 1 - time 0 hr** | 19.21 | 5.59 | 70.90 | 26.19 | 2.62 |
| **Control 2 - time 0 hr** | 8.93 | 2.69 | 69.88 | 17.30 | 1.73 |
| **Control 1 - time 3 hr** | 14.27 | 3.35 | 76.52 | 4.55 | 0.46 |
| **Control 2 - time 3 hr** | 10.03 | 2.42 | 75.87 | 33.81 | 3.38 |
| **Control 1 - time 6 hr** | 23.25 | 5.18 | 77.72 | 22.60 | 2.26 |
| **Control 2 - time 6 hr** | 11.62 | 3.16 | 72.81 | 26.15 | 2.62 |
| **Control 1 - time 12 hr** | 12.75 | 3.62 | 71.61 | 8.92 | 0.89 |
| **Control 2 - time 12 hr** | 28.29 | 6.41 | 77.34 | 9.33 | 0.93 |
| **Control 1 - time 24 hr** | 32.46 | 7.77 | 76.06 | 11.03 | 1.10 |
| **Control 2 - time 24 hr** | 9.61 | 2.24 | 76.69 | 9.16 | 0.92 |
|  |  |  |  |  |  |
| **1IU 1 - time 0 hr** | 12.23 | 3.84 | 68.60 | 34.30 | 3.43 |
| **1IU 2 - time 0 hr** | 15.08 | 4.57 | 69.69 | 31.68 | 3.17 |
| **1IU 1 - time 3 hr** | 21.57 | 6.33 | 70.65 | 6.37 | 0.64 |
| **1IU 2 - time 3 hr** | 9.80 | 2.75 | 71.94 | 1.68 | 0.17 |
| **1IU 1 - time 6 hr** | 24.69 | 6.57 | 73.39 | 2.15 | 0.21 |
| **1IU 2 - time 6 hr** | 15.65 | 3.97 | 74.63 | 11.38 | 1.14 |
| **1IU 1 - time 12 hr** | 23.95 | 6.48 | 72.94 | 2.70 | 0.27 |
| **1IU 2 - time 12 hr** | 21.97 | 5.70 | 74.06 | 3.49 | 0.35 |
| **1IU 1 - time 24 hr** | 32.32 | 7.27 | 77.51 | 1.62 | 0.16 |
| **1IU 2 - time 24 hr** | 24.94 | 6.15 | 75.34 | 2.31 | 0.23 |
|  |  |  |  |  |  |
| **0.5IU 1 - time 0 hr** | 19.10 | 5.67 | 70.31 | 16.45 | 1.64 |
| **0.5IU 2 - time 0 hr** | 18.53 | 4.43 | 76.09 | 8.15 | 0.82 |
| **0.5IU 1 - time 3 hr** | 42.42 | 5.30 | 87.51 | 1.30 | 0.13 |
| **0.5IU 2 - time 3 hr** | 20.15 | 4.34 | 78.46 | 0.75 | 0.08 |
| **0.5IU 1 - time 6 hr** | 39.54 | 9.57 | 75.80 | 3.26 | 0.33 |
| **0.5IU 2 - time 6 hr** | 27.18 | 4.52 | 83.37 | 1.75 | 0.17 |
| **0.5IU 1 - time 12 hr** | 21.10 | 5.27 | 75.02 | 2.63 | 0.26 |
| **0.5IU 2 - time 12 hr** | 14.74 | 2.40 | 83.72 | 2.11 | 0.21 |
| **0.5IU 1 - time 24 hr** | 27.74 | 6.02 | 78.30 | 1.45 | 0.14 |
| **0.5IU 2 - time 24 hr** | 33.52 | 5.38 | 83.95 | 4.37 | 0.44 |
|  |  |  |  |  |  |
| **0.25IU 1 - time 0 hr** | 14.58 | 4.88 | 66.53 | 17.35 | 1.73 |
| **0.25IU 2 - time 0 hr** | 23.26 | 6.61 | 71.58 | 21.36 | 2.14 |
| **0.25IU 1 - time 3 hr** | 17.55 | 4.77 | 76.64 | 3.50 | 0.35 |
| **0.25IU 2 - time 3 hr** | 15.69 | 3.76 | 74.89 | 3.32 | 0.33 |
| **0.25IU 1 - time 6 hr** | 20.28 | 4.10 | 76.48 | 1.91 | 0.19 |
| **0.25IU 2 - time 6 hr** | 14.26 | 3.94 | 73.63 | 2.63 | 0.26 |
| **0.25IU 1 - time 12 hr** | 18.35 | 3.53 | 80.76 | 1.11 | 0.11 |
| **0.25IU 2 - time 12 hr** | 15.73 | 3.36 | 78.64 | 0.94 | 0.09 |
| **0.25IU 1 - time 24 hr** | 30.30 | 4.40 | 85.48 | 0.95 | 0.10 |
| **0.25IU 2 - time 24 hr** | 20.13 | 4.06 | 79.83 | 0.69 | 0.07 |

Table S2: Length values in mm of individual cranial cruciate ligaments (CCLs) at different planes. Abbreviations: S1-R, specimen one of the right pelvic limb; S1-L, specimen one of the left pelvic limb; SD, standard deviation and CV, coefficient of variation.

| **Control** | | | | | | | |
| --- | --- | --- | --- | --- | --- | --- | --- |
|  | **Cranial (mm)** | **Caudal (mm)** | **Medial (mm)** | **Lateral (mm)** | **Mean (mm)** | **SD (mm)** | **CV (%)** |
| S1-R | 23.86 | 9.07 | 17.63 | 17.74 | 17.08 | 6.08 | 36 |
| S2-R | 20.51 | 11.31 | 13.6 | 15.61 | 15.26 | 3.92 | 26 |
| S3-R | 20.04 | 10.92 | 18.72 | 13.66 | 15.84 | 4.28 | 27 |
| S4-R | 23.96 | 13.19 | 19.29 | 20.57 | 19.25 | 4.50 | 23 |
| S5-R | 20.71 | 11.27 | 11.88 | 14.47 | 14.58 | 4.31 | 30 |
| S6-R | 19.49 | 10.9 | 14.76 | 19.16 | 16.08 | 4.07 | 25 |
|  |  |  |  |  |  |  |  |
| **Treated (PG depleted)** | | | | | | | |
|  | **Cranial (mm)** | **Caudal (mm)** | **Medial (mm)** | **Lateral (mm)** | **Mean (mm)** | **SD (mm)** | **CV (%)** |
| S1-L | 21.97 | 10.8 | 18.54 | 16.08 | 16.85 | 4.70 | 28 |
| S2-L | 21.88 | 13.76 | 16.85 | 18.18 | 17.67 | 3.36 | 19 |
| S3-L | 19.76 | 11.3 | 15.44 | 16.83 | 15.83 | 3.52 | 22 |
| S4-L | 21.92 | 14.38 | 15.61 | 16.29 | 17.05 | 3.34 | 20 |
| S5-L | 22.45 | 11.52 | 15.52 | 18.04 | 16.88 | 4.58 | 27 |
| S6-L | 19.95 | 11.51 | 14.03 | 13.22 | 14.68 | 3.67 | 25 |

Table S3: Cross-sectional areas (CSA) of individual canine cranial cruciate ligaments (CCLs). Abbreviations: S1-R, specimen one of the right pelvic limb; S1-L, specimen one of the left pelvic limb.

| **Control** | | **Treated (PG depleted)** | |
| --- | --- | --- | --- |
|  | **CSA (mm^2^)** |  | **CSA (mm^2^)** |
| S1-R | 20.14 | S1-L | 29.21 |
| S2-R | 25.80 | S2-L | 31.57 |
| S3-R | 24.05 | S3-L | 20.81 |
| S4-R | 20.43 | S4-L | 28.92 |
| S5-R | 17.98 | S5-L | 16.07 |
| S6-R | 29.29 | S7-L | 21.24 |
